# The Impact of Automated Electronic Surveillance of Electronic Medical Records on Pediatric Inpatient Care

**DOI:** 10.7759/cureus.3395

**Published:** 2018-10-01

**Authors:** Jais Emmanuel, Adalberto Torres

**Affiliations:** 1 Pediatrics, University of Central Florida, Orlando, USA; 2 Pediatrics, Nemours Children's Hospital, Orlando, USA

**Keywords:** electronic medical record, pediatrics, inpatient, automated, surveillance, hospitalized children, intervention, alert, retrospective study

## Abstract

Purpose

To assess the impact of the automated surveillance of the electronic medical record process on clinical interventions among hospitalized children at a tertiary care pediatric center.

Methods

A retrospective chart review of the alerts triggered for central line-associated blood stream infections (CLABSIs), catheter-associated urinary tract infections (CAUTIs), neonatal sepsis, or clinical deterioration through elevated pediatric early warning scores (PEWS) by automated electronic surveillance of the hospital electronic medical record (EMR) over a five-month period among hospitalized children. Interventions instituted in response to the alerts were reviewed from the hospital EMR. Fisher’s exact test was performed to detect any significant difference in the proportion of interventions performed for alerts triggered between groups.

Results

A total of 244 alerts were collected (27 CAUTI, 55 CLABSI, 10 neonatal sepsis, and 152 PEWS alerts). A significant difference in the proportion of interventions instituted after neonatal sepsis and PEWS alerts (9/162, 5.6%) as compared to CLABSI and CAUTI alerts (20/82, 24.4%) was observed (p<0.001; Odds ratio (95% CI): 0.182 (0.079-0.422)). Neonatal sepsis triggered the least number of alerts (10/244, 4.1%) and proportionately fewer interventions than the other clinical alerts.

Conclusions

Alerts for potential device-associated infections resulted in more clinical intervention than less-specific alerts. Neonatal sepsis alerts resulted in minimal interventions undertaken in response to the alert. Identifying and focusing on alerts benefitting the patient can serve as a better allocation of time and resources. Future studies should explore which newer alerts and their accompanying interventions improve patient outcomes.

## Introduction

Hospital-acquired infections pose an increased burden for patients and the healthcare system. Arterial catheters or central lines, while often necessary for the management of critically ill children, increase the risk of acquiring bloodstream infections [1-2]. Not only are these infections associated with increased morbidity and mortality but also result in increased healthcare cost and length of hospital stay [3-5]. A study that analyzed the 2008 to 2011 Nationwide Inpatient Sample (NIS) databases concluded that among pediatric patients with central line-associated blood stream infections (CLABSIs), the difference in the mean attributable cost and the length of stay for these patients as compared to patients without a CLABSI were $55,646 and 19 days, respectively [4]. Preventing these secondary infections can have a significant impact on patients and the healthcare system.

Automated electronic surveillance for identifying potential nosocomial infections offers more efficiency than manual surveillance [6-8]. Shepard et al. showed that detecting catheter-associated urinary tract infections (CAUTIs) utilizing an automated electronic algorithm reduced the surveillance requirement by 97.01% [6]. Traditional means of surveillance were time-consuming and resource-intensive. The study concluded that the increased efficiency offered by the automated electronic surveillance to detect healthcare-acquired infections should allow for more time to be spent on implementing appropriate interventions and improving patient care [6]. Another study that assessed the effectiveness of a fully automated surveillance system to detect intensive care unit-acquired infections found that electronic surveillance (sensitivity of 87% and specificity of 99%) was more effective than manual surveillance (sensitivity of 40% and specificity of 94%) and required fewer personnel resources by reducing the personnel time spent on surveillance by up to 85% (82.5 hours spent for manual surveillance versus 12.5 hours for electronic surveillance) [8]. While prior studies have shown the efficiency of the automated electronic approach in the surveillance of healthcare-associated infections, there are few data addressing the impact of such surveillance on the care of the patient.

At our institution, an automated electronic surveillance tool was implemented in 2012 to aid clinicians in detecting hospital-acquired infections, sepsis, or rapid clinical deterioration among hospitalized children. The automated surveillance system continuously monitored the Electronic Medical Record (EMR) of our pediatric inpatients to alert the clinician for the following: CLABSI, CAUTI, neonatal sepsis, and patients at risk for rapid clinical deterioration reflected by elevated pediatric early warning Scores (PEWS). CLABSI is defined by the presence of a laboratory-confirmed bloodstream infection after >2 calendar days of the placement of a central line or umbilical catheter and still in place on the date of the event of infection or removed the day before the event [9]. CAUTI describes patients who presented with fever and a positive bacterial culture of urine while an indwelling urinary tract catheter was in place [10]. Neonatal sepsis is the clinical syndrome caused by bacterial invasion of the bloodstream during the first month of life [11]. Since the immune system of neonates is functionally immature, neonatal sepsis can be rapidly fatal if left untreated or if the provision of antibiotic therapy is delayed [11]. PEWS is an objective scoring tool based on behavior, cardiovascular and respiratory status, nebulizer use, and a persistent post-surgical vomiting of the pediatric patient [12]. PEWS has been used to detect early changes in a patient’s physiological status so that appropriate and early interventions can be implemented for patients at risk of rapid critical deterioration. PEWS served as an early indicator of clinical deterioration for >11 hours prior to a code blue event in one study, forewarning the care team to respond to the change in status and possibly averting a code [13]. The automated surveillance system was enacted at our institution to assist in preventing potentially adverse outcomes.

This retrospective chart review was conducted to determine the impact of the automated electronic surveillance for CLABSIs, CAUTIs, neonatal sepsis, and PEWS alerts on the care of the patient by assessing the interventions instituted in response to the alerts. We postulated that this study should provide insight into which alerts were effective and necessary to be monitored and which alerts may be modified or removed due to a lack of patient benefit. We hypothesized that alerts triggered for neonatal sepsis and PEWS would have a higher proportion of interventions taken by the clinical staff compared to alerts triggered for CLABSIs and CAUTIs, as neonatal sepsis and PEWS could lead to rapid clinical deterioration if timely interventions were not performed [11,13-16].

## Materials and methods

Study design

Our hospital, a freestanding, 100-bed pediatric tertiary care center in Southeastern US, housed a specialized department known as the Clinical Logistics Center (CLC). Staffed by paramedics with expertise in pediatric emergency medicine for 24 hours a day, seven days a week, the CLC served to ensure patient safety by monitoring the digital input, e.g., patient vital signs, EMR, and patient location, of all admitted patients of ages 0 to 18 years from a remote location. The CLC utilized the EPIC Monitor software program to conduct continuous automated electronic surveillance of the EMR (Epic Hyperspace, Epic Systems Cor., Verona, Wisconsin, USA) in real time of all patients admitted to inpatient units, pediatric and neonatal intensive care units, as well as the cardiac critical care unit. Customized clinical rules were followed by the surveillance system to alert for CAUTIs, CLABSIs, neonatal sepsis, or elevated PEWS (see Appendix).

The trigger of an alert for a CLABSI, CAUTI, neonatal sepsis, or PEWS displayed a color-coded score on a monitor for the paramedics to view. The paramedics were then instructed to follow a protocol-driven response to ensure that the patient received appropriate care. For example, when a CLABSI was triggered, the EPIC Monitor displayed a score of 0, 1, 2, or 3 if the patient satisfied the criteria set by the customized clinical rules. A CLABSI score of 3 was triggered if the patient had a central venous catheter (central line), a documented fever of 100.4˚F or greater, and an elevated white blood cell count. A score of 0 was displayed if the patient did not have a central line in place or if the patient met the criteria for a CLABSI score of 3 but was being treated with appropriate antibiotics. No responses were taken by the paramedics for a CLABSI score of 0. Patients were placed on a watch list if a score of 1 or 2 was displayed and were monitored for score escalations. A score of 3 prompted the paramedic to further triage the alert. The alert was not only updated on the patient’s EMR for access to the patient’s attending physician but the physician was also immediately notified via text message in the event that appropriate antibiotics were not in place for the patient at risk for infection [17]. Similarly, other customized clinical rules were used to alert for CAUTIs, neonatal sepsis, or PEWS, and alert-specific protocol-driven responses were followed by the paramedics to further triage the alerts.

This retrospective study was approved by the local institutional review board. The database platform, Qlik Sense (v.2.2, QlikTech International AB, Lund, Sweden), was used to record and track the alerts triggered by Epic Monitor starting on October 13, 2015. The total alerts triggered from October 13, 2015, to the start of the study on March 11, 2016, were reviewed. However, only the alerts that satisfied the criteria for physician notification and documentation in the EMR were extracted for further investigation. The sample size, therefore, consisted of all the physician-notified alerts that occurred within the five-month time period. The patient information obtained from each alert was used to retrospectively review the EMR to identify the interventions instituted within a two-hour time frame after the alert onset. The two-hour window was selected to ensure that any interventions not immediately documented were included. The outcomes measured consisted of any clinical interventions, such as a diagnostic test, therapy change, or transfer to a higher level of care, instituted within the two-hour time period after the trigger of a CLABSI, CAUTI, neonatal sepsis, or PEWS alert. We excluded any interventions implemented prior to the alert onset since we wanted to ensure that the alerts themselves resulted in a change in clinical practice. Each patient’s reason for admission was also recorded. The collected data was extracted to an electronic spreadsheet on a password-protected computer, and any patient identifiers were deleted from the electronic spreadsheet after the completion of the data analysis.

Statistical analysis

Statistical analyses were conducted using the software SPSS 23.0 (IBM Corp., Armonk, NY, US). Fisher’s exact test was performed to assess the association between the categorical variables alert type (PEWS/neonatal sepsis and CLABSI/CAUTI) and intervention instituted (yes or no). Alert and intervention frequency, as well as the type of interventions instituted for individual alert and the patient’s reason for admission, are reported in frequency and percentage. Confidence interval and odds ratio are reported. All tests were two-tailed. An alpha level of 0.05 was considered statistically significant.

## Results

A total of 244 alerts triggered by Epic Monitor were notified to the attending physician and documented in the EMR from October 13, 2015, to March 11, 2016. Twenty-nine interventions resulted from the total 244 alerts (29/244, 12%). Of these, 162/244 (66.4%) alerts were recorded for neonatal sepsis and PEWS and 82/244 (33.6%) alerts were identified with CLABSI and CAUTI. There was a significantly (p < 0.001) lower proportion of interventions instituted after neonatal sepsis and PEWS alerts (9/162, 5.6%) as compared to interventions instituted after CLABSI and CAUTI alerts (20/82, 24.4%) (Odds ratio (95% CI): 0.182 (0.079-0.422)).

**Figure 1:**
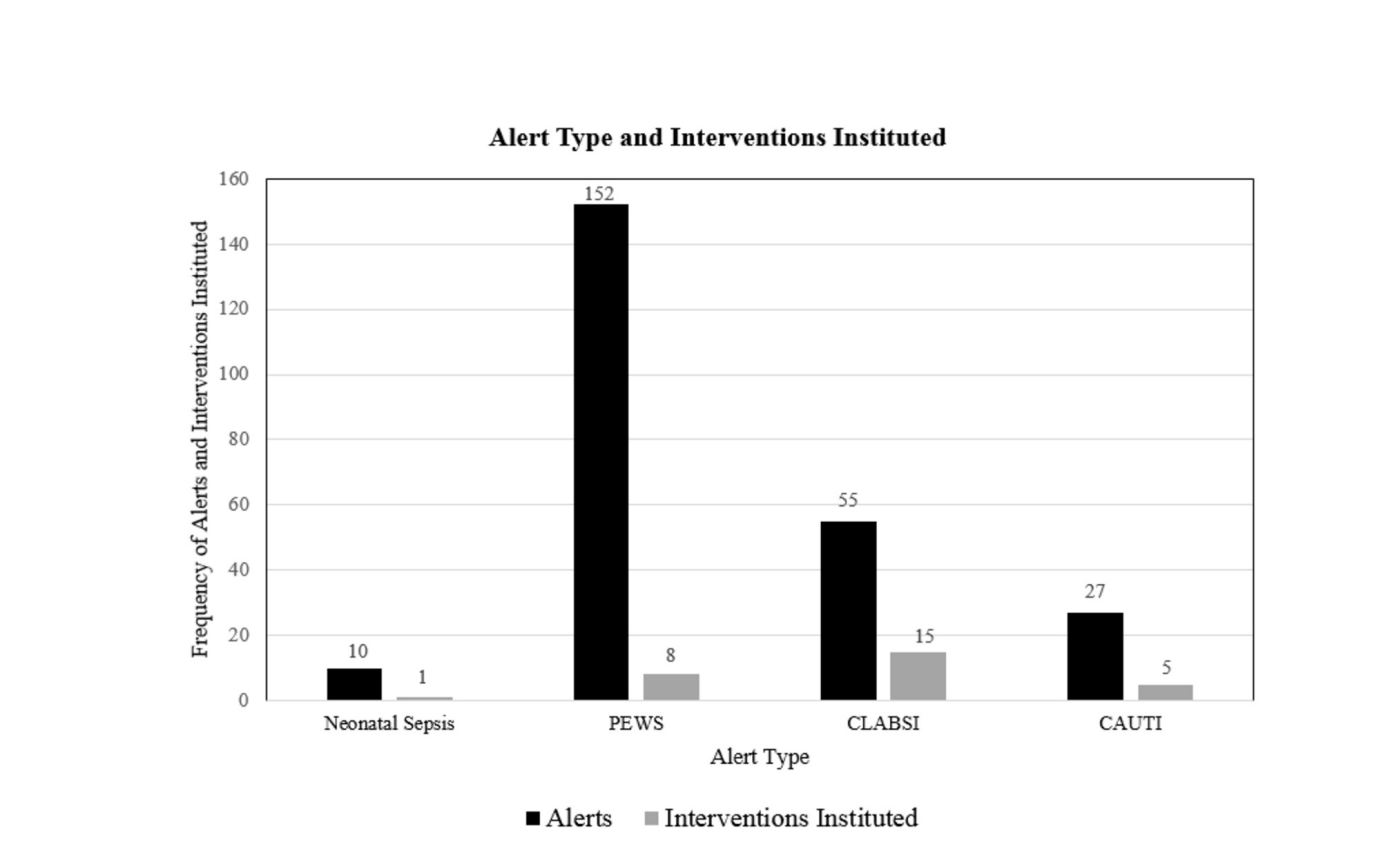
Frequency of the alerts triggered and the resulting interventions

CLABSI alerts produced the highest frequency of interventions. PEWS was the most frequently triggered alert (152/244, 62%). Neonatal sepsis alerts were the least triggered and resulted in the lowest proportion of interventions as compared to the other clinical alerts studied (Figure 1). Fifteen out of 55 (27%) CLABSI alerts resulted in diagnostic and therapeutic interventions, predominantly blood culture and antibiotic administration. And, 6/8 (75%) of PEWS interventions consisted of respiratory therapeutic interventions (Table 1). Patients who activated the CLABSI alert were most often hematology/oncology patients (30/55, 54%). PEWS alerts were often triggered by patients with respiratory conditions (Table 2).

**Table 1:** Type of intervention instituted in response to each alert Types of interventions instituted in response to each alert. Data are shown in frequency and percentage. Pediatric early warning scores (PEWS); Central line-associated blood stream infection (CLABSI); Catheter-associated urinary tract infection (CAUTI)

Type of Intervention Instituted in Response to Each Alert				
	Neonatal Sepsis	PEWS	CLABSI	CAUTI
Respiratory intervention	0	6	0	0
Transfer to higher level of care	0	1	0	0
Respiratory intervention & transfer to higher level of care	0	1	0	0
Antibiotics issued	1	0	4	1
Blood culture	0	0	2	0
Blood culture and antibiotics issued	0	0	8	2
Blood culture & antibiotics issued & transfer to higher level of care	0	0	1	0
Removal of catheter	0	0	0	2
Total interventions	1 (3%)	8 (28%)	15(52%)	5 (17%)

**Table 2:** Triggered alerts and reason for admission. Reason for hospital admission of the patients who triggered the alerts. Data are shown in frequency and percentage. Pediatric early warning scores (PEWS); Central line-associated blood stream infection (CLABSI); Catheter-associated urinary tract infection (CAUTI)

Triggered Alerts and Reason for Admission				
Neonatal Sepsis	PEWS	CLABSI	CAUTI
Hematology/Oncology	0 (0%)	16 (10%)	30 (54%)	2 (7%)
Respiratory	6 (60%)	116 (76%)	13 (24%)	1 (4%)
Gastrointestinal	1 (10%)	3 (2%)	0 (0%)	1 (4%)
Infectious Disease	3 (30%)	1 (1%)	4 (7%)	0 (0%)
Neurology	0 (0%)	4 (3%)	2 (4%)	2 (7%)
Other	0 (0%)	12 (8%)	6 (11%)	21 (78%)
Total alerts	10	152	55	27

## Discussion

CLABSI and CAUTI alerts resulted in a significantly higher proportion of interventions than neonatal sepsis and PEWS alerts. We hypothesized that neonatal sepsis and PEWS alerts would result in a higher proportion of interventions based on the severity of the conditions associated with these alerts and the potentially rapid clinical deterioration that may occur should there be a delay in interventions [11,13-16].

CLABSI alerts, by themselves, resulted in the highest proportion of interventions compared to interventions instituted after CAUTI, PEWS, and neonatal sepsis alerts combined. Blood cultures, antibiotic administration, or transfer to a higher level of care were often performed after a CLABSI alert was notified to the physician. The interventions generated by a CLABSI alert were most commonly implemented for hematology-oncology patients; typically, the immunocompromised patient with a central venous catheter in place. Clinicians seemed to be cautious of the increased susceptibility to sepsis from a central line infection in these patients. In effect, the onset of a CLABSI alert would yield a high probability of a diagnostic or therapeutic intervention in these patients to prevent the occurrence of sepsis.

PEWS objectively scores the pediatric patient based on behavior, cardiovascular and respiratory status, nebulizer use, and persistent post-surgical vomiting to identify patients who are at risk of clinical deterioration [12]. Yet, out of 152 PEWS alerts, only 8/152 (5%) alerts resulted in an intervention after the alert onset. Patients who triggered the PEWS alert were often admitted for respiratory conditions such as status asthmaticus. Clinicians seemed to be aware of the deteriorating condition of the patient even before the alert onset, as appropriate interventions were already in place or were being considered in addition to the careful and frequent monitoring of the patient prior to the activation of the alert.

Neonatal sepsis was the least triggered alert and resulted in proportionately fewer interventions than CLABSI, CAUTI, and PEWS alerts. Patients who triggered neonatal sepsis alerts were often carrying a diagnosis of a respiratory illness, such as bronchiolitis, and monitored frequently. Since neonatal sepsis alerts notified to the physician resulted in few patient care interventions, identifying and focusing on other types of clinical alerts may be more beneficial to the patient. For example, abnormal heart rate characteristics, such as reduced heart rate variability and decelerations up to 24 hours preceding an abrupt clinical deterioration from neonatal sepsis, has demonstrated to provide important and more timely information than clinical laboratory values [18-20]. In fact, a continuous monitoring of heart rate characteristics was associated with reduced sepsis-related mortality among very low birth weight infants [21]. Developing a clinical alert for neonatal sepsis utilizing the continuous automated surveillance of heart rate characteristics could potentially provide more benefit to the patient and lead to an earlier diagnosis and prevention of neonatal sepsis [18-22].

Although this study was able to shed some light on the alerts that prompted interventions, several limitations have been identified that may be resolved in future studies. First, interventions documented within the two-hour time period may not have been in response to an alert and may have been placed independently. Also, the duration of this retrospective pilot study was limited to a five-month time period during the peak season of respiratory syncytial virus (RSV)/influenza infections. Patients were frequently admitted for respiratory distress, which may have influenced the larger proportion of PEWS alerts triggered during this time period. Another potential limitation was that data were only collected at a single center. Evaluating similar automated clinical alerts may elicit different results at another institution with different volumes and types of patients.

While only 12% of the total alerts resulted in interventions in this study, studies have shown that despite the growth of medical technology, approximately 5% of patient alarms actually become clinically relevant [23-24]. Additional studies reviewing the clinical circumstances of the non-actionable alerts can contribute to a further improvement of the clinical criteria set for the alerts. Furthermore, physician surveys to gauge the clinician’s perceptions toward the alerts can help understand the contribution of alarm burden and alarm fatigue to the alert response. There is definitely an increased interest in incorporating fully automated surveillance to detect healthcare-acquired infections and early signs of clinical deterioration [6-8,25-26]. Comparing alerts and interventions in other pediatric inpatient healthcare institutions that incorporate automated electronic medical record surveillance as well as analyzing their patient care outcomes would result in the ability to collect a more robust sample of data that can help identify the type of alerts greatly impacting the care of the patient.

## Conclusions

Alerts of potential device-associated infections (CLABSI and CAUTIs) resulted in significantly more clinical interventions than less-specific alerts (neonatal sepsis and PEWS). Future studies comparing newer alerts and assessing patient care interventions may provide valuable information to clinicians on the alerts that should continue to be monitored and can improve the patient's clinical outcome.

## Figures and Tables

**Figure 2:**
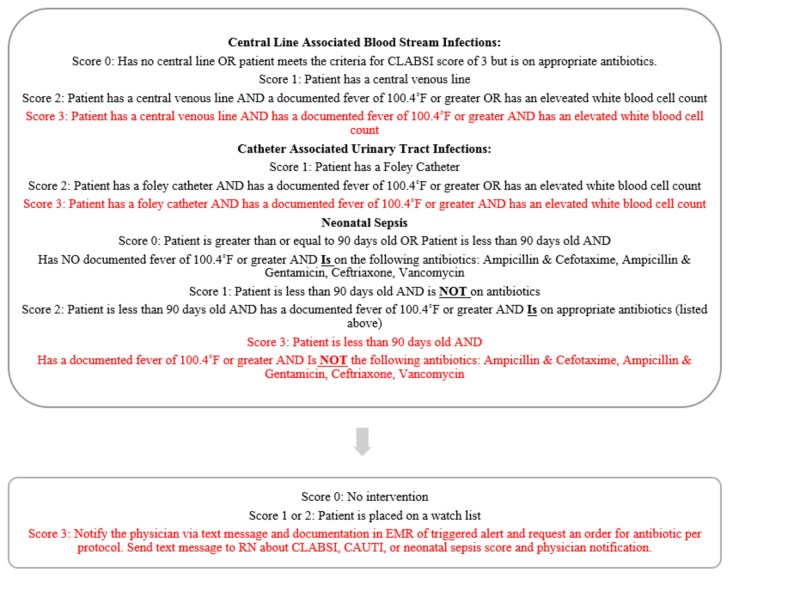
Customized EPIC Monitor rules for central line-associated blood stream infection (CLABSI), catheter-associated urinary tract Infection (CAUTI), and neonatal sepsis alerts with the corresponding action implemented by the paramedics within the Clinical Logistics Center (CLC).

**Figure 3:**
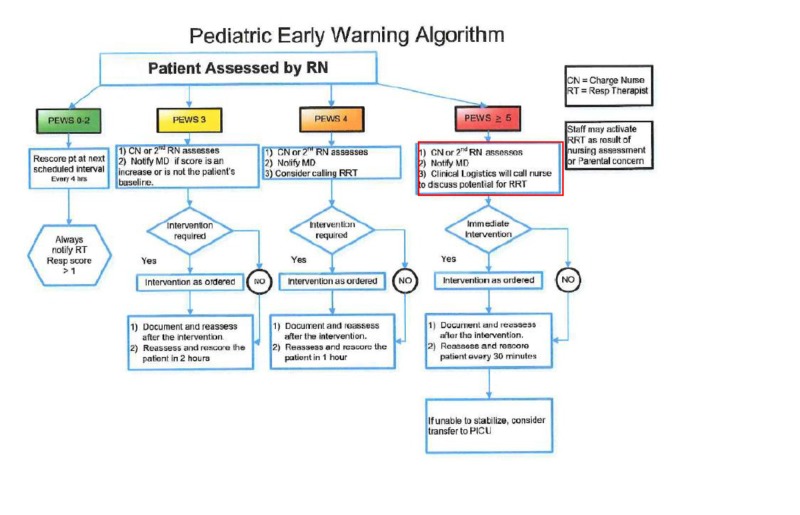
Customized pediatric early warning scores algorithm indicating the actions implemented by the healthcare team based on pediatric early warning scores (PEWS)

## Appendices

Figures 2-3 provide detailed information on the customized rules that trigger a CLABSI, CAUTI, neonatal sepsis, or PEWS alert and the specific protocol response. 
